# How Are Pedagogical Practices Within Medical Education Being Adapted for Autistic Students?

**DOI:** 10.7759/cureus.99817

**Published:** 2025-12-22

**Authors:** Alex Harker, Suhail Tarafdar

**Affiliations:** 1 General Internal Medicine, Aneurin Bevan University Health Board, Cardiff, GBR; 2 Warwick Medical School, University of Warwick, Coventry, GBR

**Keywords:** autism, inequity, medical education, pedagogical practices, strategies

## Abstract

Autism can be defined as a neurodevelopmental learning difficulty characterised by deficits in social communication, the presence of restrictive interests and repetitive behaviours. The aim of this systematic review was to explore pedagogical practices and identify interventions that would have an impact on autistic medical students' experiences.

A search strategy was undertaken on databases relevant to medical education. Included studies pertained to autistic medical students and/or postgraduate autistic doctors and concerned factors that could have implications on pedagogical practice. A quality appraisal was conducted, and a narrative synthesis was employed to produce the final report.

Seven articles were included in the final synthesis, with three deemed high risk of bias. Four themes were identified. Findings that improved experiences included raising understanding and awareness through training, recognition and language usage and individualised practical adaptations. Emphasis was placed on role modelling from autistic medical educators/clinicians to facilitate insight into strengths and weaknesses. Taking an alternative view on empathy and considering co-creation of empathy teaching sessions (faculty and autistic students) would positively impact not only autistic medical students but also their neurotypical peers.

Potential strategies have been proposed to bolster the effectiveness and equitable nature of current pedagogical practice in medical education. The more tenable propositions suggested include altering language use and co-creation of empathy teaching sessions. The remaining proposals may not currently be plausible within the United Kingdom given cost and feasibility factors when considering their implementation. Further evaluation is needed when considering the global context. The paucity of literature pertaining to this topic indicates that further research is warranted.

## Introduction and background

Medical institutions are finding themselves burdened by increasing capacity strain due to a mismatch between supply and demand: patient need outweighing physician capability [1,2]. The United Kingdom (UK) National Health Service (NHS) Long Term Workforce Plan [3] outlines key principles to increase the workforce.

One pertinent area is the ever-expanding neurodiverse medical student population [4]. The definition of neurodiversity stems from the social model of disability, which argues that neurodevelopmental differences are a normal variation and should not be discriminated against [5]. Neurodiversity includes specific learning difficulties (SpLD), which are conditions that can have a significant impact within education, the workplace environment and social situations; these include autism spectrum disorder (ASD), attention deficit hyperactivity disorder (ADHD), dyslexia and dyscalculia.

Statistics indicate the number of medical students declaring SpLD has risen from 1.4% to 4.6% between 2002 and 2018 [4], which is likely to be an underestimation as data were gathered prior to the NHS Long Term Workforce Plan implementation. An increase in medical students should correlate with an increase in SpLD students. Furthermore, students are seemingly apprehensive about declaring SpLD as they fear it will be perceived as a means to excuse poor performance [6] or because they do not wish to be identified with the associated negative perceptions attributed to neurodiversity [7,8]. Medical professionals also fear that by disclosing their condition, they would be considered disabled, therefore jeopardising their ability to practice [9].

There is growing concern that individuals are experiencing inequity within medical education due to established practices inadequately addressing neurodiverse learning needs. Greater inclusion of neurodiverse students within higher education should be commended, but there remain fundamental questions surrounding equity and whether appropriate teaching adaptations cater for these individuals. Neurodiverse individuals face numerous barriers. These can result in masking, a term used to describe neurodiverse individuals consciously or subconsciously suppressing their characteristics in order to blend in [10]. Masking has a significant impact on cognitive load [11] and therefore reduces focus on understanding key concepts. Traditional teaching modalities can be ineffectual for some neurodiverse individuals. Sumner et al. [12] found that students with SpLD demonstrate lower confidence in verbalisation. Therefore, their ability to fully comprehend topics may be diminished due to their reduced confidence in asking questions to better understand. It is important to explore how pedagogical practices are being adapted to cater for the growth of medical students with SpLD and hence to mitigate the impact of inequity.

The breadth of SpLD is considerable. Consequently, this review will focus on adaptations for autistic medical students. ASD can be defined as a disorder that is characterised by deficits in social communication, the presence of restrictive interests and repetitive behaviours [13]. However, ASD is a spectrum, and solely focusing on severe ASD can be detrimental to adaptations.

The reason for focusing on autistic medical students is due to the fact that there have been a 787% rise in the diagnosis of ASD between 1998 and 2017 [14] and, more significantly, a doubling in the percentage of people who have a diagnosis of autism without learning difficulty between 2017 and 2023 [15]. There has also been substantial negative media and literary attention on ASD, resulting in skewed public perception, more so than other SpLD, with media representation of autistic individuals often inaccurate [16] and often portrayed as unstable or dangerous [17,18]. Literature suggests that autistic individuals lack empathy due to deficits in social interaction and communication [19,20].

Whilst research has been conducted and attempts have been made to correct these inaccurate notions, they continue to be present within medical education. Thus, there is a considerable risk that pedagogical practice has not been sufficiently accommodated to reduce the inequity that autistic medical students face. It is to be noted that certain current pedagogical practices may be effective for both autistic and non-autistic individuals.

The aim of this literature review is to explore pedagogical practices and identify interventions that would have an impact on autistic students' experiences.

## Review

Methods

To collate and review the available research, a systematic approach was undertaken. This allowed for a rigorous and comprehensive assessment of the available data to identify appropriate concepts that inform practices in medical education [21,22]. 

Eligibility Criteria

Studies were only included if they pertained to autistic medical students and/or postgraduate autistic doctors and concerned areas that could have implications on pedagogical practice. It was not necessary for the studies to suggest an adjustment/catering of practice for learning needs as current teaching methods could be suitable for both autistic and non-autistic students/doctors. Postgraduate doctors were included so that feedback on what would have improved experiences at medical school was captured. Given the paucity of research into this topic, there was no timeframe limitation. This permitted comparison between historic and recent studies to evaluate changes in practice. Studies unavailable in the English language and non-primary articles were excluded.

Information Sources

Searches were undertaken in March 2025 on PubMed, Embase, Ovid MEDLINE and Google Scholar. These databases were selected because of their relevance to healthcare and medical education.

Search Strategy

The search terms were selected utilising Boolean terms and the ECLIPSE (Expectation, Client, Location, Impact, Professionals and SErvice) methodology. The ECLIPSE framework [23] (Table 1) was utilised due to the question being orientated to qualitative data.

**Table 1:** Use of the ECLIPSE framework for the research question ECLIPSE: Expectation, Client, Location, Impact, Professionals and SErvice

ECLIPSE framework	
Expectation	Determine pedagogical methods to aid autistic medical students' learning
Client	Autistic medical students
Location	Medical schools
Impact	Highlight strategies to aid autistic medical students within medical education and mitigate inequity
Professionals	Medical educators/medical schools
SErvice	Undergraduate medical education

Table 2 illustrates the search strategy undertaken.

**Table 2:** Search strategy undertaken

Search strategy
Autism OR ASD OR autis* OR SpLD OR (learning difficulties)
AND
(Medical education) OR (clinical education) OR (medical student education) OR (medical teaching) OR (postgraduate education) OR (medical school) OR (medical institution)
AND
(Medical students) OR (trainee doctors) OR (trainee physicians) OR doctors OR physicians OR (resident doctors)
AND
Experiences OR perceptions OR views OR teaching OR learning OR strategy

Refer to Appendix 1 for further search strategy information and considerations.

Data Selection and Management

Titles and abstracts of papers were screened and excluded using the eligibility criteria. Full-text reviews of the remaining studies were undertaken, and those which met the inclusion criteria were included.

Risk of Bias

Each paper that met the inclusion criteria was quality appraised as part of the systematic review to assess the risk of bias. Quality appraisal was performed through utilising the Critical Appraisal Skills Programme (CASP) with individual toolkits utilised for qualitative and cross-sectional studies [24] depending on the study being appraised.

Results

Study Selection

**Figure 1:**
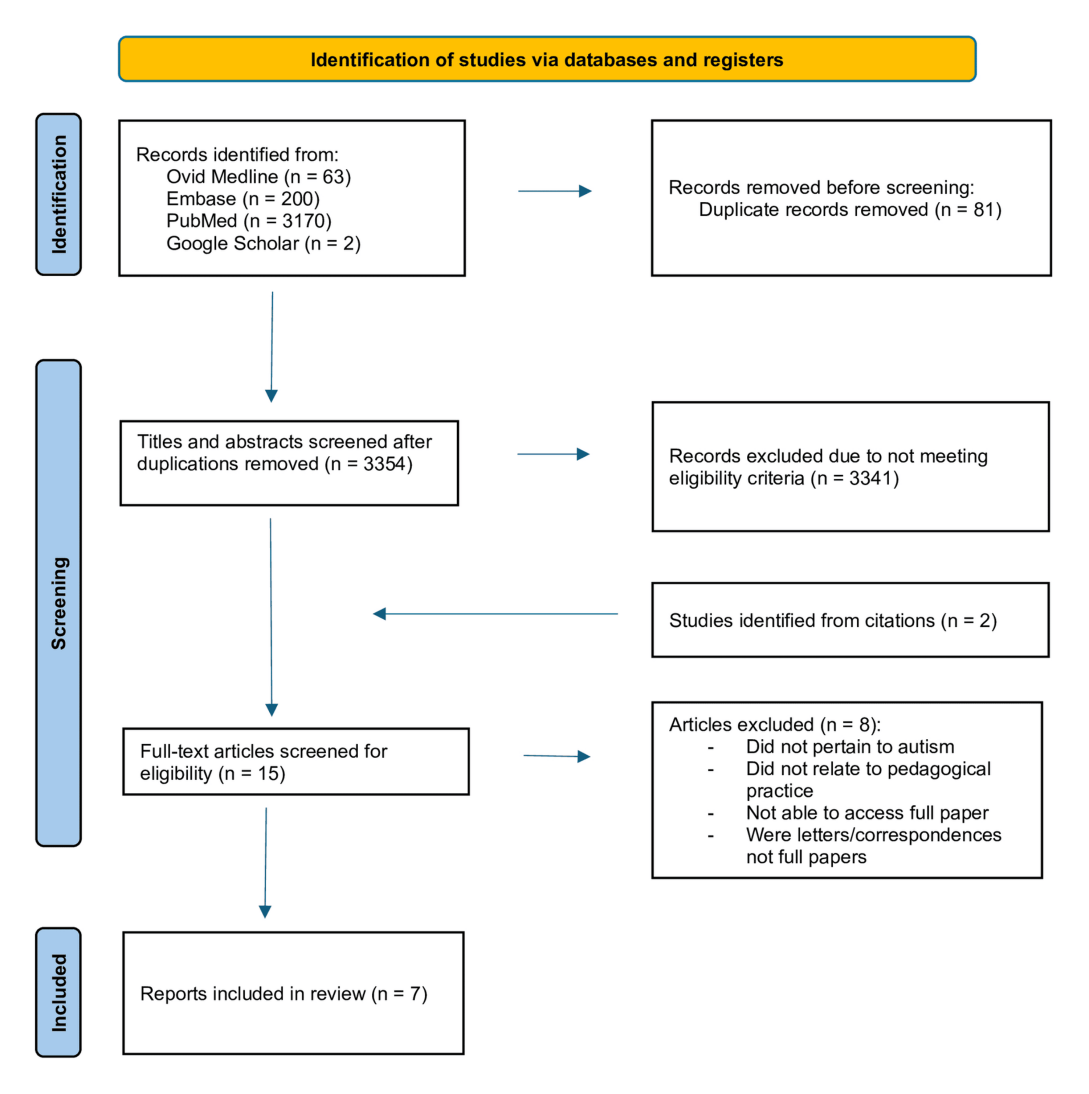
PRISMA diagram documenting the identification and screening process PRISMA: Preferred Reporting Items for Systematic Reviews and Meta-Analyses

Post-removal of duplications, the search strategy identified 3354 articles. Of those articles, 3341 were excluded during title and abstract screening for not meeting the eligibility criteria. Two further studies were identified from citations. Upon full-text review, eight of the remaining 15 articles were excluded. Of those excluded, one did not pertain to autism, three did not relate to pedagogical practice, two were letters/correspondences, and access was unobtainable to the full papers for two articles. Seven articles were included in the final narrative synthesis. Figure 1 illustrates the Preferred Reporting Items for Systematic Reviews and Meta-Analyses (PRISMA) diagram [25] outlining the identification and screening process.

Screening Process

It is to be noted that the screening process primarily involved one reviewer and the reasoning for this was due to resource constraints, particularly time. This does increase the risk of bias and human error and is subsequently a limitation of this review. However, to counteract this risk, a clear research question was constructed, and strict inclusion/exclusion criteria were established to prevent subjective post hoc decisions being made. Peer review was also performed on the search strategy to ensure its comprehensiveness. Articles that were selected and the limited articles that broadly met the inclusion criteria but had limitations were discussed amongst two reviewers in order to fully agree on inclusion. 

Characteristics of the Included Studies

Four papers were qualitative, two were cross-sectional studies, and one paper used a mixed-methods design (qualitative and cross-sectional). The characteristics of the included studies are summarised in Appendix 2.

Five papers originated from the UK, whilst two were from France. Two papers explored the experiences of autistic medical students and doctors [26,27], one delved into contextual examples coupled with explanatory concepts [28], one focused on empathy [29], one looked at medical students' attitudes towards support for disability in medicine [30], one explored the impact of an Asperger's diagnosis on doctors and education [31], and the last surveyed the opinions of medical teachers regarding students with neurodevelopmental disorders and their management [32].

Asperger's syndrome/disorder was removed from the Diagnostic and Statistical Manual of Mental Disorders Fifth Edition (DSM-5) by the American Psychiatric Association and is now subsumed under the broader diagnosis of ASD [33]. This allows the study by Price et al. [31] to be eligible.

Data Extraction

Data extraction was conducted using a template to capture preliminary interpretations as well as core points illustrated within the articles. Discussions were held between two researchers, and an iterative process was undertaken, allowing for the refinement of categories. A limitation of the data extraction process was that no software was used.

Risk of Bias From the Included Studies

A quality appraisal was undertaken of five qualitative and three cross-sectional designs, with one of these being mixed-method (qualitative and cross-sectional) and therefore appraised twice.

The CASP checklists (qualitative and cross-sectional) [24] allowed for the critical appraisal of key aspects of each study. However, CASP checklists [24] do not formally delineate between high-, moderate- and low-risk studies through the use of a scoring system; instead, they guide the user to formulate their own decisions by working through prompts [34]. 

Table 3 illustrates cut-offs for overall risk.

**Table 3:** Risk criteria

Risk criteria	
High risk	≥1 high-risk area
Moderate risk	≥2 moderate-risk areas
Low risk	<2 moderate-risk areas

Appendix 3 and Appendix 4 summarise the risk of bias appraisal for all study types and provide further details on how delineation was undertaken. Three studies were low risk, one was moderate risk, and three were high risk.

The majority of findings from high-risk papers were corroborated by other studies included within this review. When considering the face validity, the appropriateness and relevance the items of a particular measurement have to the respondents [35], in the context of the high-risk studies, two could be deemed to be flawed as they do not quantitatively or qualitatively measure any outcomes. However, given that face validity is a surface-level impression, upon reviewing these studies, an argument can be made that they do have a degree of face validity as they seemingly address the subject matter at hand and are understandable from both lay and expert perspectives. 

Moderate- and high-risk studies were included in the review as it was felt that these studies provided unique insights into key aspects that could be influencing the educational experience of autistic medical students. These included insights into the pedagogical practice surrounding empathy education [29] alongside a greater exploration of the role that autism education and recognition play within medical education [28]. The inclusion of these studies allows for a nuanced understanding of teaching practices that would otherwise have been overlooked. These studies may also support or highlight deficiencies in low-risk studies, allowing for a more rigorous exploration of this research topic.

Narrative Synthesis

A predominantly inductive approach was taken to the narrative synthesis wherein reviewers immersed themselves in the articles identified from the screening process. Comparisons were made between the articles selected, and this allowed for the development of new insights to be formed as well as the consolidation of pre-existing insights. 

Findings

There were four major themes identified having the potential to, or have, an impact on autistic medical student education. All themes are centred around pedagogical practice with the scope varying from centring on individual educators to institutional practices. No statistical synthesis was conducted due to the incompatibility of outcomes and designs.

Raised understanding and awareness improve experiences: Within this theme, there were two sub-themes: training and recognition and language use. Autistic individuals are stigmatised because of a lack of knowledge or misunderstanding by their peers/educators [36]. This perpetuates a feeling of reluctance from autistic individuals to disclose their diagnosis, therefore not receiving the support they may require [37].

Training and recognition increase understanding: Training for medical educators in ASD has a triple-fold positive impact. Studies suggest that increasing educator knowledge allows for a better understanding of ASD and leads to a reduction in negative pre-conceived views teachers may have (stemming from literature and media) which will mitigate the impact stigma has on these students [26,30].

Moreover, through the development of understanding, educators will be able to adapt their practices to provide a greater degree of personalised management [32] and subsequently will be able to better support students in developing their own insight into their diagnosis [31].

The third positive outcome of increasing understanding surrounding ASD and particularly its manifestations is that it will allow medical educators to build on their pedagogical arsenal by being in a position where they may be able to detect autistic traits/presentations in previously undiagnosed students [28].

Altering language use empowers students: There is growing evidence that incorporating a neurodiversity-affirmative approach, defined as celebrating and appreciating the unique profile of each neurodiverse individual [38], has a beneficial impact on mental health [27] as well as acknowledging the strengths that these individuals can bring to society and the workplace [39].

Shaw et al. [26] propose that, by adopting identity-first language (IFL), it empowers autistic individuals to "embrace their differences without the appendage of disorder".

Practical adaptations need to be made according to individual needs: Shaw et al. [26] found that adjustments offered were generic and led to students having to make decisions resulting in greater personal stress.

Expanding the proposed adaptation regarding developing personalised management plans [32], one proposal is providing individuals with reasonable adjustments suited to them following consultation with supervisors and faculty. Magnin et al. [32] and Shaw et al. [27] develop this further by suggesting adjustments that lead to more flexibility, greater supervision and career pathway adaptations. Two of those would involve input from medical educators (adapting current supervision practice and shifting to careers counselling which may be outside their current scope of practice).

Positive role modelling results in greater appreciation of strengths: Miller et al. [30] describe the potential adverse effect that negative role modelling, in the form of viewing conditions such as ASD as weaknesses, can have on students. They also propose that an explanation for these negative attitudes stems from the "hidden curriculum" which is surmised by Mackin et al. [40] as "a set of influences that function at the level of the organisational structure and culture to impact learning". This is further bolstered by Shaw et al. [26] who propose that the non-autistic state of being was synonymous with professionalism, thus propagating increased levels of masking from autistic students.

An adaptation to institutional pedagogical practice proposed by Giroux and Pélissier-Simard [28] and supported by Shaw et al. [41] is that a promotion of discussions between autistic medical students and autistic doctors should be advocated.

It is also argued that strong role modelling from non-autistic educators has a similar, albeit less powerful, effect. The simple switch away from the deficit-based view of autism is an effective starting point [42], further emphasising the importance of adapting understanding and knowledge.

The double empathy problem could change current educational practices: There has been a significant recent argument regarding autistic individuals and their ability or rather inability to demonstrate empathy [19,20]. Findings from Milton [29] and Shaw et al. [26] show an alternative angle in which they describe the role the double empathy problem plays. A hypothesis propagated from research into the double empathy problem suggests that neurodiverse individuals, particularly autistic individuals, have developed a greater level of insight into neurotypical society and make a greater effort into understanding emotions and feelings of neurotypical individuals.

Discussion

Implications

A recent review by Aitken et al. [43] corroborated findings found in this review regarding the importance of enhancing awareness and advocating for changes around reasonable adjustments. However, this review goes beyond their findings by highlighting key areas pertaining to language use and the role the double empathy hypothesis has. This review also expands further on the implications and limitations of role modelling, begins to consider global contexts and focuses more on pedagogical adaptations and feasibility. 

This review has identified four overarching themes, with one theme divided into sub-themes. It has found that a shift in understanding and awareness has a powerful impact on pedagogical practice regarding autistic medical students alongside having a significant role in other proposed adaptations. Considering the role stigma plays, the development of educator understanding lessens the influence of negative pre-conceived views, subsequently allowing for a stronger psychologically safe environment, a determinant that Maslow [44] argued was imperative in being able to eventually achieve self-actualisation. This shift will also facilitate the detection of characteristics displayed by undiagnosed autistic students. Early detection may be pivotal in ensuring the learning potential of these students is optimised [45].

The adoption of a more positive stance on ASD is a view that is already being advocated amongst other SpLDs. A systematic review looking into the experiences of dyslexic students and doctors reported that raised awareness would not only promote inclusivity but also reduce stigma [46]. This proposition is a supplemental adaptation to medical educators' practice as opposed to changing/overhauling current methods. Ways that could promote a more positive stance are the development of workshops and peer support programmes.

Through utilising IFL, it can be argued that the subsequent empowerment autistic students feel would mitigate the role masking has within the classroom and workplace settings, allowing a greater percentage of their focus to be on learning as opposed to fitting in. This switch in language also aims to further balance the inequity these individuals face in terms of opportunities and serves as a form of social justice. Whilst the review has identified the positive impact IFL can have, there's still conflict between person-first language (PFL) and IFL. Vivanti [47] describes that PFL was used to put the person before the disability as a means to emphasise the person's strengths unrelated to the disability. Supporting this review's findings, Taboas et al. [48] found that autistic adults overwhelmingly preferred IFL to PFL. However, Gernsbacher [49] writes that IFL and PFL can both perpetuate stigma; interestingly, the support for PFL in this article is centred within American professional organisations. Both Taboas et al. [48] and Gernsbacher [49] are American articles demonstrating the conflict within a singular country. This raises the question of whether global contexts need to be considered before PFL or IFL is proposed internationally. Further research is needed within varying countries, and there's a potential that neurodiverse individuals in one country may prefer PFL, whilst their peers in other countries may choose IFL. 

Concerning practical adaptations, this needs to be considered at both the UK and global levels. Autistic medical students in the UK are entitled to reasonable adjustments in line with the UK Equality Act [50]. This is similarly the case in America with the Americans with Disabilities Act [51]. Apart from the UK and America, certain countries are disadvantaged as legislation is inadequate and, as such, supportive provisions are limited [52]. The ability to change these stances is grounded in developing a greater understanding of ASD. The implementation of personalised reasonable adjustments would demonstrate to the autistic student that they are being listened to and their concerns are being addressed. This would alleviate stress and foster a psychologically sound environment. This implementation would also reflect on the institutions with neurodiverse students developing greater confidence in the medical school(s) subsequently opening up more with faculty breaking the opinion these individuals may have on the institutions seeing them as disabled. However, there lies a paradox concerning the use of the term "disability" with this cohort. Under the current legislation, having a disability label may be fundamental for the provision of adjustments. Therefore, the greater the confidence these students have in the institutions, the greater the chance of shifting the negative connotation of disability to one of recognition of a student's strengths and weaknesses. 

Within medical education, positive role modelling from medical educators/clinicians and subsequent observation of best practice have a greater effect on learning than normal teaching [53]. Discussions between autistic students and autistic doctors will enable students to have a greater degree of insight into their strengths and vulnerabilities and consequently may be the difference between continuing their career in medicine or seeking an alternative vocation, thus providing the UK, from a retention perspective, with a Long Term Workforce Plan.

The impact the double empathy hypothesis has had on pedagogical practice is currently unknown but likely to be minimal at this stage. It does, however, suggest an adaptation to empathy education and, in the broader sense, communication skills. The current assumption, and traditional teaching method, is that neurotypical educators teach neurodiverse students regarding empathy. An adaptation would be to take a collaborative approach to empathy teaching with autistic students, providing an in-depth insight into their practices, which might in turn enrich the education of their neurotypical peers.

Limitations

Considering the limitations of the proposed adaptations, it is worth considering these in the context of an adjusted utility equation [54], with the utility equation being $\mathrm{Utility}=\mathrm{Reliability}\times\mathrm{Validity}\times\text{Educational Impact}\times\mathrm{Acceptability}\times\mathrm{Cost}$. It was proposed that an assessment cannot fulfil each element perfectly and therefore evaluating each element permits appropriate compromises to be made [55]. Replacing "reliability" and "validity" with "feasibility" allows greater evaluation of the proposals outlined within this review. The adapted utility equation would subsequently be portrayed as follows: $\text{Adapted Utility}=\mathrm{Feasibility}\times\text{Educational Impact}\times\mathrm{Acceptability}\times\mathrm{Cost}$.

Evaluating the theme regarding understanding and awareness, it is apparent that from an educational impact and acceptability (all stakeholders) stance, this is a strong proposition. In terms of feasibility, there has been no elaboration on the method to provide this education to educators. No specific information has been provided on whether the education will be in the form of didactic teaching, online learning or another form. The method employed will be a key determinant in the cost associated with this adaptation subsequently having a significant impact on the practicality of its implementation. There is also the hidden cost associated with educators using their time to undertake this learning. It can be argued that medical educators have a degree of internal motivation driving increased understanding. However, it is naïve to assume this would fully outweigh the external motivation of financial gain.

Methods to address these challenges would be leveraging resources and utilising existing teaching strategies, e.g. the National Autistic Society provides training resources designed to increase understanding and support mechanisms.

When considering practical adaptations, limitations could be argued within the categories of feasibility and cost. Within the UK, a key determinant is the impact of the Long Term Workforce Plan. There is no obvious emphasis on increasing the number of medical educators in proportion to the increasing medical student population/incentivising being an educator. This conundrum, together with the proposal for more personalised management plans, significantly and detrimentally impacts the feasibility of this suggestion. The suggestion that current medical educators should also formally act as career counsellors may not be within their current scope of educational practice. This will involve greater investment in educating educators, together with time to construct appropriate teaching resources to allow these sessions to take place. Both factors are limited by resources; therefore, this proposal may not be practically or economically feasible within the current UK climate.

Global considerations should be evaluated; whereas, in the UK, there are financial constraints, the financial model employed in America may allow greater flexibility for clinicians to take on these roles. This is an area that needs greater focus, particularly when comparing medical education practices on a global scale. 

Compared to previous proposals, the suggestion regarding utilising autistic doctors/medical educators as positive role models is less confined by cost and feasibility but by acceptability. There is considerable stigma surrounding autism, particularly amongst medical professionals, as they believe that the association it has with disability would jeopardise their ability to practice [9]. This unfortunately contributes to a perpetuating cycle whereby autistic medical educators do not want to jeopardise their careers and hence do not wish to undertake any role modelling. The lack of role modelling prohibits autistic students from developing insights into their strengths and weaknesses; thus, they either leave the profession or avoid role modelling themselves when they graduate due to the fear of stigma. The only current suggestion for breaking this cycle is increased understanding and awareness around autism.

Contrasting the limitations of other suggestions, the proposal pertaining to the co-creation of empathy teaching sessions is arguably the most balanced, requiring no considerable increase in cost to establish, has good acceptability from all stakeholders, has a strong educational impact and is practically feasible. Considerable research exists outlining the benefits of co-creation from the aspect of both building positive relationships between faculty and students [56] and increasing student satisfaction and enhancing personal development within higher education [57].

Adapting the utility equation allows for direction and focus. A caveat to consider is that it has been established for evaluating assessments as opposed to adaptations of pedagogical practice. Assessment evaluation considers quantitative aspects, whereas a significant portion of the articles reviewed were qualitative in nature. Adjusting the Utility Equation could limit its efficacy and may not be the most appropriate mechanism for evaluating the proposals.

Study Limitations

There is a paucity of literature pertaining to this topic. Moreover, of those studies included, three were deemed high risk. There is also a lack of global representation, with the studies included being from two European countries (the UK and France). Further research is therefore required.

## Conclusions

Taking into consideration the points illustrated in this review, there are a number of implications for practice identified. The first is that raising understanding and awareness aids in destigmatisation and permits personalised management plans to be developed. There is also the fact that the majority of practical adaptations are generic and subsequently not suitable; therefore, developing individualised reasonable adjustments, albeit resource-heavy, will improve student experiences. Another area identified is the potential positive impact co-creation of empathy sessions (faculty and autistic students) could have, and, subsequently, further research should be conducted on the outcomes of these sessions.

Utilising IFL may empower students. However, there is no current consensus on IFL versus PFL; therefore, using them interchangeably may be advocated until more definitive research is conducted. Further research is also indicated comparing medical education systems worldwide to establish barriers to interventions and elicit ways to overcome them.

## Appendices

Appendix 1

**Table 4:** Search strategies conducted using various databases using Boolean logic When developing the search strategy, it was initially proposed to include OR between ((medical education) OR (clinical education) OR (medical student education) OR (medical teaching) OR (postgraduate education) OR (medical school) OR (medical institution)) and ((medical students) OR (trainee doctors) OR (trainee physicians) OR doctors OR physicians OR (resident doctors)). However, after performing this search, the refinement on the number of studies was inadequate, and, after a brief review, the studies that came up were in no way aligned to the research question at hand.

Search strategies	
PubMed search (3170 articles identified from the search)	Search: ((autism OR autis* OR SpLD OR (learning difficulties)) AND ((medical education) OR (clinical education) OR (medical student education) OR (medical teaching) OR (postgraduate education) OR (medical school) OR (medical institution)) AND ((medical students) OR (trainee doctors) OR (trainee physicians) OR doctors OR physicians OR (resident doctors)) AND ((experiences OR perceptions OR views OR teaching OR learning OR strategy) ("autism s"[All Fields] OR "autisms"[All Fields] OR "autistic disorder"[MeSH Terms] OR ("autistic"[All Fields] AND "disorder"[All Fields]) OR "autistic disorder"[All Fields] OR "autism"[All Fields] OR "autis*"[All Fields] OR "SpLD"[All Fields] OR (("learning"[MeSH Terms] OR "learning"[All Fields] OR "learn"[All Fields] OR "learned"[All Fields] OR "learning s"[All Fields] OR "learnings"[All Fields] OR "learns"[All Fields]) AND ("difficulties"[All Fields] OR "difficulty"[All Fields]))) AND ("education, medical"[MeSH Terms] OR ("education"[All Fields] AND "medical"[All Fields]) OR "medical education"[All Fields] OR ("medical"[All Fields] AND "education"[All Fields]) OR (("ambulatory care facilities"[MeSH Terms] OR ("ambulatory"[All Fields] AND "care"[All Fields] AND "facilities"[All Fields]) OR "ambulatory care facilities"[All Fields] OR "clinic"[All Fields] OR "clinic s"[All Fields] OR "clinical"[All Fields] OR "clinically"[All Fields] OR "clinicals"[All Fields] OR "clinics"[All Fields]) AND ("educability"[All Fields] OR "educable"[All Fields] OR "educates"[All Fields] OR "education"[MeSH Subheading] OR "education"[All Fields] OR "educational status"[MeSH Terms] OR ("educational"[All Fields] AND "status"[All Fields]) OR "educational status"[All Fields] OR "education"[MeSH Terms] OR "education s"[All Fields] OR "educational"[All Fields] OR "educative"[All Fields] OR "educator"[All Fields] OR "educator s"[All Fields] OR "educators"[All Fields] OR "teaching"[MeSH Terms] OR "teaching"[All Fields] OR "educate"[All Fields] OR "educated"[All Fields] OR "educating"[All Fields] OR "educations"[All Fields])) OR (("students, medical"[MeSH Terms] OR ("students"[All Fields] AND "medical"[All Fields]) OR "medical students"[All Fields] OR ("medical"[All Fields] AND "student"[All Fields]) OR "medical student"[All Fields]) AND ("educability"[All Fields] OR "educable"[All Fields] OR "educates"[All Fields] OR "education"[MeSH Subheading] OR "education"[All Fields] OR "educational status"[MeSH Terms] OR ("educational"[All Fields] AND "status"[All Fields]) OR "educational status"[All Fields] OR "education"[MeSH Terms] OR "education s"[All Fields] OR "educational"[All Fields] OR "educative"[All Fields] OR "educator"[All Fields] OR "educator s"[All Fields] OR "educators"[All Fields] OR "teaching"[MeSH Terms] OR "teaching"[All Fields] OR "educate"[All Fields] OR "educated"[All Fields] OR "educating"[All Fields] OR "educations"[All Fields])) OR (("medic"[All Fields] OR "medical"[All Fields] OR "medicalization"[MeSH Terms] OR "medicalization"[All Fields] OR "medicalizations"[All Fields] OR "medicalize"[All Fields] OR "medicalized"[All Fields] OR "medicalizes"[All Fields] OR "medicalizing"[All Fields] OR "medically"[All Fields] OR "medicals"[All Fields] OR "medicated"[All Fields] OR "medication s"[All Fields] OR "medics"[All Fields] OR "pharmaceutical preparations"[Supplementary Concept] OR "pharmaceutical preparations"[All Fields] OR "medication"[All Fields] OR "pharmaceutical preparations"[MeSH Terms] OR ("pharmaceutical"[All Fields] AND "preparations"[All Fields]) OR "medications"[All Fields]) AND ("education"[MeSH Subheading] OR "education"[All Fields] OR "teaching"[All Fields] OR "teaching"[MeSH Terms] OR "teaches"[All Fields] OR "teach"[All Fields] OR "teachings"[All Fields] OR "teaching s"[All Fields])) OR (("postgraduate"[All Fields] OR "postgraduated"[All Fields] OR "postgraduates"[All Fields] OR "postgraduation"[All Fields]) AND ("educability"[All Fields] OR "educable"[All Fields] OR "educates"[All Fields] OR "education"[MeSH Subheading] OR "education"[All Fields] OR "educational status"[MeSH Terms] OR ("educational"[All Fields] AND "status"[All Fields]) OR "educational status"[All Fields] OR "education"[MeSH Terms] OR "education s"[All Fields] OR "educational"[All Fields] OR "educative"[All Fields] OR "educator"[All Fields] OR "educator s"[All Fields] OR "educators"[All Fields] OR "teaching"[MeSH Terms] OR "teaching"[All Fields] OR "educate"[All Fields] OR "educated"[All Fields] OR "educating"[All Fields] OR "educations"[All Fields])) OR ("schools, medical"[MeSH Terms] OR ("schools"[All Fields] AND "medical"[All Fields]) OR "medical schools"[All Fields] OR ("medical"[All Fields] AND "school"[All Fields]) OR "medical school"[All Fields]) OR (("medic"[All Fields] OR "medical"[All Fields] OR "medicalization"[MeSH Terms] OR "medicalization"[All Fields] OR "medicalizations"[All Fields] OR "medicalize"[All Fields] OR "medicalized"[All Fields] OR "medicalizes"[All Fields] OR "medicalizing"[All Fields] OR "medically"[All Fields] OR "medicals"[All Fields] OR "medicated"[All Fields] OR "medication s"[All Fields] OR "medics"[All Fields] OR "pharmaceutical preparations"[Supplementary Concept] OR "pharmaceutical preparations"[All Fields] OR "medication"[All Fields] OR "pharmaceutical preparations"[MeSH Terms] OR ("pharmaceutical"[All Fields] AND "preparations"[All Fields]) OR "medications"[All Fields]) AND ("academies and institutes"[MeSH Terms] OR ("academies"[All Fields] AND "institutes"[All Fields]) OR "academies and institutes"[All Fields] OR "institute"[All Fields] OR "institutes"[All Fields] OR "health facilities"[MeSH Terms] OR ("health"[All Fields] AND "facilities"[All Fields]) OR "health facilities"[All Fields] OR "institution"[All Fields] OR "institut"[All Fields] OR "institute s"[All Fields] OR "instituted"[All Fields] OR "instituting"[All Fields] OR "institution s"[All Fields] OR "institutional"[All Fields] OR "institutions"[All Fields] OR "instituts"[All Fields]))) AND ("students, medical"[MeSH Terms] OR ("students"[All Fields] AND "medical"[All Fields]) OR "medical students"[All Fields] OR ("medical"[All Fields] AND "students"[All Fields]) OR (("trainee"[All Fields] OR "trainee s"[All Fields] OR "trainees"[All Fields]) AND ("doctor s"[All Fields] OR "doctoral"[All Fields] OR "doctorally"[All Fields] OR "doctorate"[All Fields] OR "doctorates"[All Fields] OR "doctoring"[All Fields] OR "physicians"[MeSH Terms] OR "physicians"[All Fields] OR "doctor"[All Fields] OR "doctors"[All Fields])) OR (("trainee"[All Fields] OR "trainee s"[All Fields] OR "trainees"[All Fields]) AND ("physician s"[All Fields] OR "physicians"[MeSH Terms] OR "physicians"[All Fields] OR "physician"[All Fields] OR "physicians s"[All Fields])) OR ("doctor s"[All Fields] OR "doctoral"[All Fields] OR "doctorally"[All Fields] OR "doctorate"[All Fields] OR "doctorates"[All Fields] OR "doctoring"[All Fields] OR "physicians"[MeSH Terms] OR "physicians"[All Fields] OR "doctor"[All Fields] OR "doctors"[All Fields]) OR ("physician s"[All Fields] OR "physicians"[MeSH Terms] OR "physicians"[All Fields] OR "physician"[All Fields] OR "physicians s"[All Fields]) OR (("internship and residency"[MeSH Terms] OR ("internship"[All Fields] AND "residency"[All Fields]) OR "internship and residency"[All Fields] OR "residencies"[All Fields] OR "residency"[All Fields] OR "reside"[All Fields] OR "resided"[All Fields] OR "residence"[All Fields] OR "residence s"[All Fields] OR "residences"[All Fields] OR "residency s"[All Fields] OR "resident"[All Fields] OR "resident s"[All Fields] OR "residents"[All Fields] OR "resides"[All Fields] OR "residing"[All Fields]) AND ("doctor s"[All Fields] OR "doctoral"[All Fields] OR "doctorally"[All Fields] OR "doctorate"[All Fields] OR "doctorates"[All Fields] OR "doctoring"[All Fields] OR "physicians"[MeSH Terms] OR "physicians"[All Fields] OR "doctor"[All Fields] OR "doctors"[All Fields]))) AND ("experience"[All Fields] OR "experience s"[All Fields] OR "experiences"[All Fields] OR ("percept"[All Fields] OR "perceptibility"[All Fields] OR "perceptible"[All Fields] OR "perception"[MeSH Terms] OR "perception"[All Fields] OR "perceptions"[All Fields] OR "perceptional"[All Fields] OR "perceptive"[All Fields] OR "perceptiveness"[All Fields] OR "percepts"[All Fields]) OR ("viewed"[All Fields] OR "viewing"[All Fields] OR "viewings"[All Fields] OR "views"[All Fields]) OR ("education"[MeSH Subheading] OR "education"[All Fields] OR "teaching"[All Fields] OR "teaching"[MeSH Terms] OR "teaches"[All Fields] OR "teach"[All Fields] OR "teachings"[All Fields] OR "teaching s"[All Fields]) OR ("learning"[MeSH Terms] OR "learning"[All Fields] OR "learn"[All Fields] OR "learned"[All Fields] OR "learning s"[All Fields] OR "learnings"[All Fields] OR "learns"[All Fields]) OR ("strategie"[All Fields] OR "strategies"[All Fields] OR "strategy"[All Fields] OR "strategy s"[All Fields])) Translations autism: "autism's"[All Fields] OR "autisms"[All Fields] OR "autistic disorder"[MeSH Terms] OR ("autistic"[All Fields] AND "disorder"[All Fields]) OR "autistic disorder"[All Fields] OR "autism"[All Fields] learning: "learning"[MeSH Terms] OR "learning"[All Fields] OR "learn"[All Fields] OR "learned"[All Fields] OR "learning's"[All Fields] OR "learnings"[All Fields] OR "learns"[All Fields] difficulties: "difficulties"[All Fields] OR "difficulty"[All Fields] medical education: "education, medical"[MeSH Terms] OR ("education"[All Fields] AND "medical"[All Fields]) OR "medical education"[All Fields] OR ("medical"[All Fields] AND "education"[All Fields]) clinical: "ambulatory care facilities"[MeSH Terms] OR ("ambulatory"[All Fields] AND "care"[All Fields] AND "facilities"[All Fields]) OR "ambulatory care facilities"[All Fields] OR "clinic"[All Fields] OR "clinic's"[All Fields] OR "clinical"[All Fields] OR "clinically"[All Fields] OR "clinicals"[All Fields] OR "clinics"[All Fields] education: "educability"[All Fields] OR "educable"[All Fields] OR "educates"[All Fields] OR "education"[Subheading] OR "education"[All Fields] OR "educational status"[MeSH Terms] OR ("educational"[All Fields] AND "status"[All Fields]) OR "educational status"[All Fields] OR "education"[MeSH Terms] OR "education's"[All Fields] OR "educational"[All Fields] OR "educative"[All Fields] OR "educator"[All Fields] OR "educator's"[All Fields] OR "educators"[All Fields] OR "teaching"[MeSH Terms] OR "teaching"[All Fields] OR "educate"[All Fields] OR "educated"[All Fields] OR "educating"[All Fields] OR "educations"[All Fields] medical student: "students, medical"[MeSH Terms] OR ("students"[All Fields] AND "medical"[All Fields]) OR "medical students"[All Fields] OR ("medical"[All Fields] AND "student"[All Fields]) OR "medical student"[All Fields] education: "educability"[All Fields] OR "educable"[All Fields] OR "educates"[All Fields] OR "education"[Subheading] OR "education"[All Fields] OR "educational status"[MeSH Terms] OR ("educational"[All Fields] AND "status"[All Fields]) OR "educational status"[All Fields] OR "education"[MeSH Terms] OR "education's"[All Fields] OR "educational"[All Fields] OR "educative"[All Fields] OR "educator"[All Fields] OR "educator's"[All Fields] OR "educators"[All Fields] OR "teaching"[MeSH Terms] OR "teaching"[All Fields] OR "educate"[All Fields] OR "educated"[All Fields] OR "educating"[All Fields] OR "educations"[All Fields] medical: "medic"[All Fields] OR "medical"[All Fields] OR "medicalization"[MeSH Terms] OR "medicalization"[All Fields] OR "medicalizations"[All Fields] OR "medicalize"[All Fields] OR "medicalized"[All Fields] OR "medicalizes"[All Fields] OR "medicalizing"[All Fields] OR "medically"[All Fields] OR "medicals"[All Fields] OR "medicated"[All Fields] OR "medication's"[All Fields] OR "medics"[All Fields] OR "pharmaceutical preparations"[Supplementary Concept] OR "pharmaceutical preparations"[All Fields] OR "medication"[All Fields] OR "pharmaceutical preparations"[MeSH Terms] OR ("pharmaceutical"[All Fields] AND "preparations"[All Fields]) OR "medications"[All Fields] teaching: "education"[Subheading] OR "education"[All Fields] OR "teaching"[All Fields] OR "teaching"[MeSH Terms] OR "teaches"[All Fields] OR "teach"[All Fields] OR "teachings"[All Fields] OR "teaching's"[All Fields] postgraduate: "postgraduate"[All Fields] OR "postgraduated"[All Fields] OR "postgraduates"[All Fields] OR "postgraduation"[All Fields] education: "educability"[All Fields] OR "educable"[All Fields] OR "educates"[All Fields] OR "education"[Subheading] OR "education"[All Fields] OR "educational status"[MeSH Terms] OR ("educational"[All Fields] AND "status"[All Fields]) OR "educational status"[All Fields] OR "education"[MeSH Terms] OR "education's"[All Fields] OR "educational"[All Fields] OR "educative"[All Fields] OR "educator"[All Fields] OR "educator's"[All Fields] OR "educators"[All Fields] OR "teaching"[MeSH Terms] OR "teaching"[All Fields] OR "educate"[All Fields] OR "educated"[All Fields] OR "educating"[All Fields] OR "educations"[All Fields] medical school: "schools, medical"[MeSH Terms] OR ("schools"[All Fields] AND "medical"[All Fields]) OR "medical schools"[All Fields] OR ("medical"[All Fields] AND "school"[All Fields]) OR "medical school"[All Fields] medical: "medic"[All Fields] OR "medical"[All Fields] OR "medicalization"[MeSH Terms] OR "medicalization"[All Fields] OR "medicalizations"[All Fields] OR "medicalize"[All Fields] OR "medicalized"[All Fields] OR "medicalizes"[All Fields] OR "medicalizing"[All Fields] OR "medically"[All Fields] OR "medicals"[All Fields] OR "medicated"[All Fields] OR "medication's"[All Fields] OR "medics"[All Fields] OR "pharmaceutical preparations"[Supplementary Concept] OR "pharmaceutical preparations"[All Fields] OR "medication"[All Fields] OR "pharmaceutical preparations"[MeSH Terms] OR ("pharmaceutical"[All Fields] AND "preparations"[All Fields]) OR "medications"[All Fields] institution: "academies and institutes"[MeSH Terms] OR ("academies"[All Fields] AND "institutes"[All Fields]) OR "academies and institutes"[All Fields] OR "institute"[All Fields] OR "institutes"[All Fields] OR "health facilities"[MeSH Terms] OR ("health"[All Fields] AND "facilities"[All Fields]) OR "health facilities"[All Fields] OR "institution"[All Fields] OR "institut"[All Fields] OR "institute's"[All Fields] OR "instituted"[All Fields] OR "instituting"[All Fields] OR "institution's"[All Fields] OR "institutional"[All Fields] OR "institutions"[All Fields] OR "instituts"[All Fields] medical students: "students, medical"[MeSH Terms] OR ("students"[All Fields] AND "medical"[All Fields]) OR "medical students"[All Fields] OR ("medical"[All Fields] AND "students"[All Fields]) trainee: "trainee"[All Fields] OR "trainee's"[All Fields] OR "trainees"[All Fields] doctors: "doctor's"[All Fields] OR "doctoral"[All Fields] OR "doctorally"[All Fields] OR "doctorate"[All Fields] OR "doctorates"[All Fields] OR "doctoring"[All Fields] OR "physicians"[MeSH Terms] OR "physicians"[All Fields] OR "doctor"[All Fields] OR "doctors"[All Fields] trainee: "trainee"[All Fields] OR "trainee's"[All Fields] OR "trainees"[All Fields] physicians: "physician's"[All Fields] OR "physicians"[MeSH Terms] OR "physicians"[All Fields] OR "physician"[All Fields] OR "physicians's"[All Fields] doctors: "doctor's"[All Fields] OR "doctoral"[All Fields] OR "doctorally"[All Fields] OR "doctorate"[All Fields] OR "doctorates"[All Fields] OR "doctoring"[All Fields] OR "physicians"[MeSH Terms] OR "physicians"[All Fields] OR "doctor"[All Fields] OR "doctors"[All Fields] physicians: "physician's"[All Fields] OR "physicians"[MeSH Terms] OR "physicians"[All Fields] OR "physician"[All Fields] OR "physicians's"[All Fields] resident: "internship and residency"[MeSH Terms] OR ("internship"[All Fields] AND "residency"[All Fields]) OR "internship and residency"[All Fields] OR "residencies"[All Fields] OR "residency"[All Fields] OR "reside"[All Fields] OR "resided"[All Fields] OR "residence"[All Fields] OR "residence's"[All Fields] OR "residences"[All Fields] OR "residency's"[All Fields] OR "resident"[All Fields] OR "resident's"[All Fields] OR "residents"[All Fields] OR "resides"[All Fields] OR "residing"[All Fields] doctors: "doctor's"[All Fields] OR "doctoral"[All Fields] OR "doctorally"[All Fields] OR "doctorate"[All Fields] OR "doctorates"[All Fields] OR "doctoring"[All Fields] OR "physicians"[MeSH Terms] OR "physicians"[All Fields] OR "doctor"[All Fields] OR "doctors"[All Fields] experiences: "experience"[All Fields] OR "experience's"[All Fields] OR "experiences"[All Fields] perceptions: "percept"[All Fields] OR "perceptibility"[All Fields] OR "perceptible"[All Fields] OR "perception"[MeSH Terms] OR "perception"[All Fields] OR "perceptions"[All Fields] OR "perceptional"[All Fields] OR "perceptive"[All Fields] OR "perceptiveness"[All Fields] OR "percepts"[All Fields] views: "viewed"[All Fields] OR "viewing"[All Fields] OR "viewings"[All Fields] OR "views"[All Fields] teaching: "education"[Subheading] OR "education"[All Fields] OR "teaching"[All Fields] OR "teaching"[MeSH Terms] OR "teaches"[All Fields] OR "teach"[All Fields] OR "teachings"[All Fields] OR "teaching's"[All Fields] learning: "learning"[MeSH Terms] OR "learning"[All Fields] OR "learn"[All Fields] OR "learned"[All Fields] OR "learning's"[All Fields] OR "learnings"[All Fields] OR "learns"[All Fields] strategy: "strategie"[All Fields] OR "strategies"[All Fields] OR "strategy"[All Fields] OR "strategy's"[All Fields]
Embase search (200 articles identified from the search)	Embase Classic+Embase 1 autism/ or autism.mp. 2 ASD.mp. 3 autis*.mp. 4 SpLD.mp. 5 learning difficulties.mp. or learning disorder/ 6 1 or 2 or 3 or 4 or 5 7 medical education.mp. or medical education/ 8 clinical education.mp. or clinical education/ 9 medical student education.mp. 10 medical teaching.mp. or medical education/ 11 postgraduate education.mp. or postgraduate education/ 12 medical school.mp. or medical school/ 13 medical institution.mp. 14 7 or 8 or 9 or 10 or 11 or 12 or 13 15 medical students.mp. or medical student/ 16 trainee doctors.mp. 17 trainee physicians.mp. 18 doctors.mp. or physician/ 19 physicians.mp. or physician/ 20 resident doctors.mp. or resident/ 21 15 or 16 or 17 or 18 or 19 or 20 22 experiences.mp. 23 perceptions.mp. 24 views.mp. 25 teaching.mp. or teaching/ 26 learning.mp. or learning/ 27 strategy.mp. 28 22 or 23 or 24 or 25 or 26 or 27 29 6 and 14 and 21 and 28
MEDLINE (63 articles identified from the search)	Ovid MEDLINE(R) and Epub Ahead of Print, In-Process, In-Data-Review & Other Non-Indexed Citations, Daily and Versions 1 autism/ or autism.mp. 2 ASD.mp. 3 autis*.mp. 4 SpLD.mp. 5 learning difficulties.mp. or learning disorder/ 6 1 or 2 or 3 or 4 or 5 7 medical education.mp. or medical education/ 8 clinical education.mp. or clinical education/ 9 medical student education.mp. 10 medical teaching.mp. or medical education/ 11 postgraduate education.mp. or postgraduate education/ 12 medical school.mp. or medical school/ 13 medical institution.mp. 14 7 or 8 or 9 or 10 or 11 or 12 or 13 15 medical students.mp. or medical student/ 16 trainee doctors.mp. 17 trainee physicians.mp. 18 doctors.mp. or physician/ 19 physicians.mp. or physician/ 20 resident doctors.mp. or resident/ 21 15 or 16 or 17 or 18 or 19 or 20 22 experiences.mp. 23 perceptions.mp. 24 views.mp. 25 teaching.mp. or teaching/ 26 learning.mp. or learning/ 27 strategy.mp. 28 22 or 23 or 24 or 25 or 26 or 27 29 6 and 14 and 21 and 28
Google Scholar (2 articles identified from the search)	(Autism OR ASD OR SpLD OR (learning difficulties)) AND ((medical education) OR (clinical education) OR (medical student education) OR (medical teaching) OR (postgraduate education) OR (medical school) OR (medical institution)) AND ((medical students) OR (trainee doctors) OR (trainee physicians) OR doctors OR physicians OR (resident doctors)) AND (Experiences OR perceptions OR views OR teaching OR learning OR strategy)

Appendix 2

**Table 5:** Study characteristics and main findings for each paper included in the systematic review MM: The study was a mixed-methods design incorporating both qualitative and cross-sectional methodologies. Therefore, a bias assessment has been conducted for both study methodologies.

Authors	Design	Aims	Undergraduate or postgraduate	Participant characteristics	Country	Main findings
Magnin et al. (2021) [32] (MM)	Mixed-methods: cross-sectional and qualitative	Describe medical teachers’ opinions of students with neurodevelopmental disorders and their management	Postgraduate and undergraduate	Survey sent to 175 medical teachers at the University of Lyon who were participating in a pedagogical postgraduate degree. Sixty-seven responded (38%)	France	Many medical teachers report having encountered students who might have had neurodevelopmental disorders (dyspraxia 33%; dyslexia 46%; autism spectrum disorders 68%; attention deficit hyperactivity disorders 75%). Medical teachers feel unprepared to manage students with neurodevelopmental disorders. They would be interested in specific training and procedures about the pedagogic management of these students
Shaw et al. (2023) [26]	Qualitative	Explore the experiences of autistic medical students	Undergraduate	Five students participated from five different medical schools: two males and three females with ages ranging from 22 to 27	UK	Participants longed for understanding and support from their medical schools. They reported experiences of isolation, bullying and anxiety. Most felt themselves to be victims of the system, whereby they were expected to adapt themselves in order to appear non-autistic. When participants reported not coping due to being autistic, most were advised to "take time out". None were offered personalised adjustments to their learning environment
Giroux and Pélissier-Simard (2021) [28]	Qualitative	Raise awareness and understanding of difficulties related to autistic traits	N/A	N/A	France	Autistic traits in medical learners can contribute to challenging situations in education which are often overlooked by educators. Autistic traits are associated with strengths but also pose difficulties in learning and professional interactions
Milton (2012) [29]	Qualitative	Explore the ontological status of autism and introduce the "double empathy" problem	N/A	N/A	UK	Outlines the double empathy problem. Critiques traditional autism theories and proposes an alternative view on the cause of difficulties regarding social interactions
Price et al. (2017) [31]	Qualitative	Explore the impact of receiving an Asperger syndrome diagnosis and the clarity and misunderstanding that comes with it	Postgraduate	Ten interviewees. Consisted of three trainees with a recent diagnosis of autism spectrum disorder, three case managers and four specialists working with the Professional Support Unit	UK	Receiving a diagnosis of Asperger syndrome was viewed as a double-edged sword, allowing the development of insight into lifelong difficulties, but also creating the potential for prejudice. Understanding Asperger syndrome traits provided an explanation for challenges in the workplace and therefore opportunities to find solutions
Shaw et al. (2023) [27]	Cross-sectional	Explore the experiences of autistic doctors	Postgraduate	225 respondents. 82% currently working as doctors. 64% had a formal diagnosis of autism.	UK	Autistic doctors reported many challenges in the workplace. This may have contributed to a culture of nondisclosure. Mental health was poor with high rates of suicidal ideation, self-harm and prior suicide attempts. Despite inhospitable environments, most were persevering and working successfully. Viewing autism as a disorder was associated with prior suicide attempts and a preference for person-first language. A neurodiversity-affirmative approach to autism may lead to a more positive self-identity and improved mental health
Miller et al.(2009) [30]	Cross-sectional	Examine rates and types of disability in medical students, whether students disclosed this disability and their support needs, and, if not, possible reasons for non-disclosure	Undergraduate	Survey sent to 944 medical students at the School of Medicine, University of Aberdeen. 328 responses received (35%)	UK	Non-disclosure of disability in medical students may be due to several factors, including narrow definitions of disability and negative attitudes towards disability from the wider student body

Appendix 3

**Table 6:** Risk of bias for qualitative studies utilising the CASP qualitative checklist CASP: Critical Appraisal Skills Programme Key: *: low risk; **: moderate risk; ***: high risk MM: The study was a mixed-methods design incorporating both qualitative and cross-sectional methodologies. Therefore, a bias assessment has been conducted for both study methodologies.

Authors	Research aim and design	Sampling strategy	Data collection methods	Reflexivity and bias consideration	Ethical considerations	Findings and credibility	Usefulness and application
Magnin et al. (2021) [32]	Clear statement of objective and appropriate design*	Respectable sample size*	Mixed methodology allowing for a greater depth of data*	Potential biases are acknowledged, particularly with regard to self-reported attitudes*	Ethics approval obtained and consent to participate outlined. Maintained anonymity throughout*	Content validity addressed and findings are supported by qualitative and quantitative results allowing for greater inference*	Provides insight into areas for improvement with actionable recommendations*
Shaw et al. (2023) [26]	Aim stated and appropriate design (phenomenological approach)*	Recruited five medical students from five different universities. Greater sample size could have provided a greater degree of transferability. Obtaining individuals from five different medical schools did bolster this though**	Audio-recorded interviews allowed for the in-depth exploration of experiences*	Potential biases are acknowledged. Authors acknowledge their positionalities*	Ethical approval and informed consent obtained*	An array of findings illustrated which were strengthened by direct participant quotes*	Useful at highlighting the personal nature and adjustments needed. Recommendations are slightly generic and further work is needed to make them actionable**
Giroux and Pélissier-Simard (2021) [28]	Aim is clearly stated and contextual examples coupled with explanatory concepts used*	Utilises case-based examples as opposed to empirical data which potentially lessens the strength of this study***	Case-based examples which allows for a broad discussion. Qualitative data from affected individuals would have provided greater depth***	Potential biases are acknowledged. Authors consider a variety of contributing factors*	No involvement of direct participants so ethical approval was not needed**	Theoretical insights are demonstrated. Empirical data would have provided validation and therefore strengthened this study**	Emphasises the importance of recognising struggling autistic students and provides relevant pedagogical adaptations*
Milton (2012) [29]	Exploration of the ontology of autism is stated and utilisation of known data is used*	No participants used. Critiques established research. Empirical data would have bolstered the strength of this study***	Utilises established literature allowing for generalised discussion to be had. Lacks empirical data which would have provided greater depth of discussion***	This study aimed to reframe autism spectrum disorder through the critique of established research. Limited degree of acknowledgement of biases**	No involvement of direct participants so ethical approval was not needed**	Theoretical insights are demonstrated. Empirical data would have provided validation and therefore strengthened this study**	Emphasises the importance of reframing autism spectrum disorder with particular stress on empathy. Does not provide tangible adaptations that can be brought into pedagogical practice**
Price et al. (2017) [31]	Aim stated and appropriate design*	Sampling relevant. However, size and representativeness could be further assessed**	Semi-structured interviews allow for the in-depth exploration of ideas*	Potential biases are acknowledged. However, positionalities of the authors are not directly addressed*	Ethical approval and consent are addressed. Have not published further demographic details to ensure anonymity*	Elicits both benefits and challenges. Findings are supported by direct participant quotes*	Useful study which provides relevant recommendations*

**Table 7:** Criteria to delineate between low, moderate and high risk in each section

Category	Risk definition
Research aim and design	Low risk: Clearly outlined
	Moderate risk: Outlined to a degree but some inference needed
	High risk: Not outlined
Sampling strategy	Low risk: Strong sample size taken from appropriate setting
	Moderate risk: Sample size adequate/sample taken from multiple settings to aid representativeness
	High risk: No participants used
Data collection methods	Low risk: Data collection settings and method were clear and appropriate
	Moderate risk: Data collection settings and method were to a degree appropriate but lacked certain specifics/needed elaborating upon
	High risk: Data collection method was not clear or appropriate
Reflexivity and bias consideration	Low risk: Evidence of reflexivity and bias considerations
	Moderate risk: Limited evidence of reflexivity and bias considerations
	High risk: No evidence of reflexivity or bias considerations
Ethical considerations	Low risk: Ethical considerations clearly stated
	Moderate risk: Some ethical considerations clearly stated/a degree of inference needed
	High risk: No ethical considerations discussed
Findings and credibility	Low risk: Findings are clear with consideration of their credibility/supporting data provided
	Moderate risk: Findings can be inferred/are theoretical in nature
	High risk: Findings are unclear with no supporting data provided
Usefulness and application	Low risk: Provides insight into areas for improvement/appropriate changes with actionable recommendations
	Moderate risk: Provides insight into areas for improvement/appropriate changes but does not provide means to implement these
	High risk: No actionable recommendations suggested

Appendix 4

**Table 8:** Risk of bias for cross-sectional studies utilising the CASP cross-sectional checklist CASP: Critical Appraisal Skills Programme Key: *: low risk; **: moderate risk; ***: high risk MM: The study was a mixed-methods design incorporating both qualitative and cross-sectional methodologies. Therefore, a bias assessment has been conducted for both study methodologies.

Authors	Validity		Results			Applicability	
	Study aim	Sample strategy	Presentation	Alignment with the research question	Confounding factors	Ethical considerations	Application
Magnin et al. (2021) [32] (MM)	Clearly outlined*	Reasonable sample size with an approximate 38% response rate*	Findings clearly laid out. Mixed methodology with quantitative and qualitative results appropriately displayed*	Good alignment between the research question and the results obtained*	Acknowledges potential factors influencing responses. The study did not explore the role institutions may have on pedagogical practices (amenability to change teaching methods, etc.)**	Ethics approval obtained and consent to participate outlined. Maintained anonymity throughout*	Provides insight into areas for improvement with actionable recommendations. Worth noting this study did not expressively comment on the variability between institutions and the impact this might have*
Shaw et al. (2023) [27]	Clearly outlined*	Good sample size: 225 responses. There may be a degree of response bias*	Findings clearly laid out. Quantitative statistics clearly laid out*	Clear link between research topic and the results obtained*	Acknowledges potential factors that may impact results. Good spread of demographic with 46% of those that participated having completed training and 82% still practicing*	Ethics approval obtained and anonymity preserved throughout*	The study is highly relevant and proposes a neurodiversity-affirmative approach. Does not provide tangible processes to integrate the findings at an institutional level**
Miller et al.(2009) [30]	Clearly outlined*	Strong sample size. However, participants were all from one university which reduces the generalisability**	Findings clearly outlined*	Clear alignment between the research aim and the results obtained*	Acknowledges factors that would influence responses including the role institutions play within the support system. The study does discuss the impact of organisational culture but greater discussion on other cultural impacts would have strengthened this paper further*	Ethics approval was run by the appropriate committee but ethics permission was not required for this project. Informed consent was obtained*	This study provides highly relevant statistical information which could be used by medical schools. However, it does not provide actionable recommendations***

**Table 9:** Criteria to delineate between low, moderate and high risk in each section

Category	Risk definition
Study aim	Low risk: Clearly outlined
	Moderate risk: Outlined to a degree but some inference needed
	High risk: Not outlined
Sample strategy	Low risk: Strong sample size taken from appropriate setting
	Moderate risk: Sample size adequate/sample taken from multiple settings to aid representativeness
	High risk: No participants used
Presentation	Low risk: Findings clearly laid out
	Moderate risk: Findings demonstrated but not in the most appropriate way
	High risk: Findings not clearly outlined/considerable inference needed to ascertain findings
Alignment with the research question	Low risk: Clear alignment between research question and findings
	Moderate risk: Debatable link demonstrated but argument could be made that findings are associated with the research question
	High risk: No tenable link between the research question and findings demonstrated
Confounding factors	Low risk: Confounding factors acknowledged
	Moderate risk: Some confounding factors acknowledged
	High risk: No discussion around confounding factors
Ethical considerations	Low risk: Ethical considerations clearly stated
	Moderate risk: Some ethical considerations clearly stated/a degree of inference needed
	High risk: No ethical considerations discussed
Application	Low risk: Provides insight into areas for improvement/appropriate changes with actionable recommendations
	Moderate risk: Provides insight into areas for improvement/appropriate changes but does not provide means to implement these
	High risk: No actionable recommendations suggested

## References

[1] Arogyaswamy S, Vukovic N, Keniston A (2022). The impact of hospital capacity strain: a qualitative analysis of experience and solutions at 13 academic medical centers. J Gen Intern Med.

[2] Eriksson CO, Stoner RC, Eden KB, Newgard CD, Guise JM (2017). The association between hospital capacity strain and inpatient outcomes in highly developed countries: a systematic review. J Gen Intern Med.

[3] (2023). NHS Long Term Workforce Plan. https://www.england.nhs.uk/wp-content/uploads/2023/06/nhs-long-term-workforce-plan-v1.21.pdf.

[4] Murphy MJ, Dowell JS, Smith DT (2022). Factors associated with declaration of disability in medical students and junior doctors, and the association of declared disability with academic performance: observational study using data from the UK Medical Education Database, 2002-2018 (UKMED54). BMJ Open.

[5] Doyle N (2020). Neurodiversity at work: a biopsychosocial model and the impact on working adults. Br Med Bull.

[6] Newlands F, Shrewsbury D, Robson J (2015). Foundation doctors and dyslexia: a qualitative study of their experiences and coping strategies. Postgrad Med J.

[7] Dwyer P (2022). The neurodiversity approach(es): what are they and what do they mean for researchers?. Hum Dev.

[8] Griffin E, Pollak D (2009). Student experiences of neurodiversity in higher education: insights from the BRAINHE project. Dyslexia.

[9] Hamilton RH, Williams ZJ, Brodkin ES (2025). Embracing neurodiversity in medicine-building a more inclusive physician workforce. JAMA.

[10] Miller D, Rees J, Pearson A (2021). "Masking is life": experiences of masking in autistic and nonautistic adults. Autism Adulthood.

[11] Syharat C, Hain A, Zaghi A, Berdanier C (2023). Burnout: the cost of masking neurodiversity in graduate STEM programs. https://par.nsf.gov/servlets/purl/10502909.

[12] Sumner E, Crane L, Hill EL (2021). Examining academic confidence and study support needs for university students with dyslexia and/or developmental coordination disorder. Dyslexia.

[13] Hodges H, Fealko C, Soares N (2020). Autism spectrum disorder: definition, epidemiology, causes, and clinical evaluation. Transl Pediatr.

[14] Russell G, Stapley S, Newlove-Delgado T (2022). Time trends in autism diagnosis over 20 years: a UK population-based cohort study. J Child Psychol Psychiatry.

[15] (2023). Health and care of people with learning disabilities, experimental statistics 2022 to 2023. Experimental Statistics.

[16] Wood C, Freeth M (2016). Students' stereotypes of autism. J Educ Issues.

[17] Huws J, Jones R (2010). 'They just seem to live their lives in their own little world': lay perceptions of autism. Disability and Society.

[18] Jones S, Harwood V (2009). Representations of autism in Australian print media. Disability and Society.

[19] (2013). Diagnostic and Statistical Manual of Mental Disorders, Fifth Edition.

[20] Morrison KE, Pinkham AE, Kelsven S, Ludwig K, Penn DL, Sasson NJ (2019). Psychometric evaluation of social cognitive measures for adults with autism. Autism Res.

[21] Cook DJ, Mulrow CD, Haynes RB (1997). Systematic reviews: synthesis of best evidence for clinical decisions. Ann Intern Med.

[22] Maggio LA, Samuel A, Stellrecht E (2022). Systematic reviews in medical education. J Grad Med Educ.

[23] Wildridge V, Bell L (2002). How CLIP became ECLIPSE: a mnemonic to assist in searching for health policy/management information. Health Info Libr J.

[24] (2023). CASP checklists. https://casp-uk.net/casp-tools-checklists/.

[25] Page MJ, McKenzie JE, Bossuyt PM (2021). The PRISMA 2020 statement: an updated guideline for reporting systematic reviews. BMJ.

[26] Shaw SC, Doherty M, Anderson JL (2023). The experiences of autistic medical students: a phenomenological study. Med Educ.

[27] Shaw SC, Fossi A, Carravallah LA, Rabenstein K, Ross W, Doherty M (2023). The experiences of autistic doctors: a cross-sectional study. Front Psychiatry.

[28] Giroux M, Pélissier-Simard L (2021). Shedding light on autistic traits in struggling learners: a blind spot in medical education. Perspect Med Educ.

[29] Milton D (2012). On the ontological status of autism: the 'double empathy problem'. Disability and Society.

[30] Miller S, Ross S, Cleland J (2009). Medical students' attitudes towards disability and support for disability in medicine. Med Teach.

[31] Price S, Lusznat R, Mann R, Locke R (2017). Doctors with Asperger's: the impact of a diagnosis. Clin Teach.

[32] Magnin E, Ryff I, Moulin T (2021). Medical teachers' opinions about students with neurodevelopmental disorders and their management. BMC Med Educ.

[33] Gamlin C (2017). When Asperger's disorder came out. Psychiatr Danub.

[34] Long H, French D, Brooks J (2020). Optimising the value of the critical appraisal skills programme (CASP) tool for quality appraisal in qualitative evidence synthesis. Research Methods in Medicine and Health Sciences.

[35] Engel L, Bucholc J, Mihalopoulos C, Mulhern B, Ratcliffe J, Yates M, Hanna L (2020). A qualitative exploration of the content and face validity of preference-based measures within the context of dementia. Health Qual Life Outcomes.

[36] Mazumder R, Thompson-Hodgetts S (2019). Stigmatization of children and adolescents with autism spectrum disorders and their families: a scoping study. Rev J Autism Dev Disord.

[37] Turnock A, Langley K, Jones CR (2022). Understanding stigma in autism: a narrative and theoretical model. Autism Adulthood.

[38] Lerner MD, Gurba AN, Gassner D (2023). A framework for neurodiversity-affirming interventions for autistic individuals. J Consult Clin Psychol.

[39] Hartman D, Day A, O’Donnell-Killen O’Donnell-Killen, Doyle J, Kavanagh M and Azevedo J (2024 (2024). What does it mean to be neurodiversity affirmative?. https://www.bps.org.uk/psychologist/what-does-it-mean-be-neurodiversity-affirmative.

[40] Mackin R, Baptiste S, Niec A, Kam AJ (2019). The hidden curriculum: a good thing?. Cureus.

[41] Shaw SC, Grosjean B, McCowan S, Kinnear M, Doherty M (2022). Autistic role modelling in medical education. Educ Prim Care.

[42] Shaw SC, Doherty M, McCowan S, Davidson IA (2022). Challenging the exclusion of autistic medical students. Lancet Psychiatry.

[43] Aitken D, Hodge G, Page M, Lastmann E, Hiller S, George RE (2025). Navigating medical school with autism: a systematic review exploring student experiences & support provision in the United Kingdom. BMC Med Educ.

[44] Maslow A (1987). Motivation and Personality. https://www.holybooks.com/wp-content/uploads/Motivation-and-Personality-Maslow.pdf.

[45] Okoye C, Obialo-Ibeawuchi CM, Obajeun OA (2023). Early diagnosis of autism spectrum disorder: a review and analysis of the risks and benefits. Cureus.

[46] Tarafdar SA, Seoudi N, Luo R, Winston K (2025). Experiences of medical students and doctors with dyslexia: a systematic review. Med Educ.

[47] Vivanti G (2020). Ask the editor: what is the most appropriate way to talk about individuals with a diagnosis of autism?. J Autism Dev Disord.

[48] Taboas A, Doepke K, Zimmerman C (2023). Preferences for identity-first versus person-first language in a US sample of autism stakeholders. Autism.

[49] Gernsbacher MA (2017). Editorial perspective: the use of person-first language in scholarly writing may accentuate stigma. J Child Psychol Psychiatry.

[50] United Kingdom Government (2010 (2010). Equality Act 2010. Equality Act.

[51] (1990). The Americans with Disabilities Act (ADA) protects people with disabilities from discrimination. https://www.ada.gov/#:~:text=The%20Americans%20with%20Disabilities%20Act%20%20protects%20people%20with%20disabilities,many%20areas%20of%20public%20life.

[52] Majumder MA, Rahman S, D’Souza UJ, Elbeheri G, Abdulrahman KB, Huq MM (2010). Supporting medical students with learning disabilities in Asian medical schools. Adv Med Educ Pract.

[53] Benbassat J (2014). Role modelling in medical education: the importance of a reflective imitation. Acad Med.

[54] Van Der Vleuten CP (1996). The assessment of professional competence: developments, research and practical implications. Adv Health Sci Educ Theory Pract.

[55] van der Vleuten CP, Schuwirth LW (2005). Assessing professional competence: from methods to programmes. Med Educ.

[56] Bovill C (2020). Co-creation in learning and teaching: the case for a whole-class approach in higher education. High Educ.

[57] Lubicz-Nawrocka T (2018). Students as partners in learning and teaching: the benefits of co-creation of the curriculum. International Journal for Students as Partners.

